# Fusion FISH Imaging: Single-Molecule Detection of Gene Fusion Transcripts *In Situ*

**DOI:** 10.1371/journal.pone.0093488

**Published:** 2014-03-27

**Authors:** Fatu Badiane Markey, William Ruezinsky, Sanjay Tyagi, Mona Batish

**Affiliations:** 1 Department of Microbiology and Molecular Genetics, New Jersey Medical School, Rutgers University, Newark, New Jersey, United States of America; 2 Public Health Research Institute, New Jersey Medical School, Rutgers University, Newark, New Jersey, United States of America; CCR, National Cancer Institute, NIH, United States of America

## Abstract

Double-stranded DNA breaks occur on a regular basis in the human genome as a consequence of genotoxic stress and errors during replication. Usually these breaks are rapidly and faithfully repaired, but occasionally different chromosomes, or different regions of the same chromosome, are fused to each other. Some of these aberrant chromosomal translocations yield functional recombinant genes, which have been implicated as the cause of a number of lymphomas, leukemias, sarcomas, and solid tumors. Reliable methods are needed for the *in situ* detection of the transcripts encoded by these recombinant genes. We have developed just such a method, utilizing single-molecule fluorescence *in situ* hybridization (sm-FISH), in which approximately 50 short fluorescent probes bind to adjacent sites on the same mRNA molecule, rendering each target mRNA molecule visible as a diffraction-limited spot in a fluorescence microscope. Utilizing this method, gene fusion transcripts are detected with two differently colored probe sets, each specific for one of the two recombinant segments of a target mRNA; enabling the fusion transcripts to be seen in the microscope as distinct spots that fluoresce in both colors. We demonstrate this method by detecting the BCR-ABL fusion transcripts that occur in chronic myeloid leukemia cells, and by detecting the EWSR1-FLI1 fusion transcripts that occur in Ewing's sarcoma cells. This technology should pave the way for accurate *in situ* typing of many cancers that are associated with, or caused by, fusion transcripts.

## Introduction

Chromosomal aberrations, including deletions, inversions, and translocations, which occur as the result of genotoxic stress, sometimes lead to the development of cancer [1], [2]. The most common type of chromosomal aberration associated with cancer development is chromosomal translocation, which involves the rearrangement of portions of nonhomologous chromosomes, leading to the fusion of two otherwise separate genes [3]. The products of these gene fusions can cause the abnormal expression of transcription factors or the unregulated activation of tyrosine kinases, both of which lead to uncontrolled cell growth, ultimately causing cancer [4]. For many years after the initial discovery of the gene fusion responsible for chronic myeloid leukemia (CML) in 1960, gene fusions were thought to be limited to hematological malignancies [3], [5], [6]. More recently, gene fusions were found to be responsible for soft-tissue sarcomas, prostrate cancer, lung cancer, and certain solid tumors [3], [4], [6], [7]. Currently, with the advent of genome-wide translocation sequencing techniques, there is a growing list of tumors possessing an underlying gene fusion [7]–[9].

The realization that widespread gene fusions underlie many cancers has impelled the development of diagnostic assays and therapeutic drugs that target gene fusion products [10]. This has improved the quality of life, and dramatically increased the survival rate of patients, as evidenced by the marked decline in mortality associated with CML [10], [11]. Existing diagnostic assays identify gene fusions at the genomic level, using methods such as chromosomal banding, PCR, DNA fluorescence *in situ* hybridization (FISH), and deep sequencing [12]. Since treatments target the products of gene fusions, rather than the fused gene itself, genome-based methods are not direct predictors of a patient's response to therapy. Therapies directed against fusion gene products can only work if the fusion gene is active. Sensitive diagnostic assays that detect the presence of the transcripts or protein products of fused genes are needed. In the case of CML, this need is addressed by the use of real-time PCR assays that target the junction sequence in the fusion transcript [11]. This method enables the detection of fusion transcripts that may be rare compared to normal transcripts, but does not provide any information on the number of fusion mRNAs in individual cells. Also, rearrangements other than known break points and associated deletions result in different amplicons that may not amplify at the same rate, leading to inconclusive results. Although PCR is effective in finding rare cancer cells expressing fused genes in blood, it is not very useful for analyzing the distribution of cancer cells in solid and soft-tissue tumors [11], [12].

A desirable method for the detection of gene fusions, and for monitoring therapy, will provide contextual information that locates transformed cells with respect to normal cells in a tissue sample. Using current FISH methodology, this information can be obtained for individual cells, but the analysis is restricted to the detection of DNA, rather than to the detection of the much more ubiquitous fusion mRNA transcripts. A method that detects and quantifies fusion transcripts is needed to better follow disease progression and to monitor the effectiveness of therapy.

We have employed single-molecule fluorescence *in situ* hybridization (sm-FISH) to detect and quantify gene fusion transcripts. We probe two segments of the fusion transcripts with differently colored probe sets, and then image the cells in the corresponding fluorescence channels of a fluorescence microscope. Diffraction-limited spots visible in both channels correspond to individual fusion transcripts. These spots are seen only in the cancerous cells, and serve as definitive markers of these cells. This method not only permits the detection of individual fusion transcript molecules, it provides an explicit count of the number of fusion transcripts in each cell.

## Materials and Methods

### Cell lines

K562 leukemia cells (ATCC) were grown in Iscove's modified Dulbecco's medium (IMDM) supplemented with 10% fetal bovine serum (FBS) and penicillin-streptomycin (Corning cellgro). HeLa cells were cultured in Dulbecco's modified Eagle's medium with Glutamax (DMEM, Invitrogen) supplemented with 10% FBS and penicillin-streptomycin. Ewing's sarcoma cell line RD-ES was purchased from ATCC and the cell lines A673, TC71 and SK-ES-1 were obtained as a gift from Dr. James Wells at the Beth Israel Deaconess Medical Center of Harvard Medical School [13]. RD-ES and TC-71 Ewing's sarcoma cell lines were cultured in high glucose Roswell Park Memorial Institute (RPMI) 1640 medium (ATCC) supplemented with 15% FBS and penicillin-streptomycin. SK-ES-1 and A673 Ewing's sarcoma cell lines were cultured in McCoy's 5A medium (ATCC) with 15% FBS and DMEM with 10% FBS, respectively, supplemented with penicillin-streptomycin. All cell types were grown in 100 cm^2^ tissue culture dishes containing glass coverslips coated with a laminin/poly D lysine or 0.1% gelatin for adherence. All reagents were purchased from Sigma, unless specified otherwise.

### Probe synthesis and *in situ* hybridization

Sets of 48 linear oligonucleotides, each about 20 nucleotides in length, were designed to hybridize to a pre-selected region within a target mRNA (Table S1). The oligonucleotides were purchased from Biosearch Technologies with an amino group on their 3′ ends. These oligonucleotides were pooled in equimolar amounts and reacted with succinimidyl esters of the fluorophores tetramethylrhodamine (TMR) or Alexa Fluor 594. The labeled probes were purified from unlabeled probes and loose fluorophores by high-pressure liquid chromatography, as described previously [14]. Coverslips with adhered cells were washed with phosphate-buffered saline solution (PBS), fixed with 3.7% (vol/vol) formaldehyde for 10 min and permeabilized in 70% ethanol at 4°C for at least 1 hr. Following a wash with 10% formamide dissolved in saline sodium citrate solution (2× SSC, Ambion), the cells were hybridized to probe sets in 50 μL of hybridization buffer containing 10% (wt/vol) dextran sulfate (Sigma), 1 μg/μL *Escherichia coli* tRNA (Sigma), 2 mM ribonucleoside-vanadyl complex (New England Biolabs) to inhibit ribonucleases, 0.02% (wt/vol) ribonuclease-free bovine serum albumin (Ambion), 10% (vol/vol) formamide (Ambion), and 5 ng/μL of each probe set. Hybridization was done overnight at 37°C in a moist chamber. On the next day, the coverslips were washed three times with 10% formamide in 2× SSC solution, and then mounted in 2% catalase/glucose oxidase (Sigma) containing mounting media, as described previously [14], [15]. When utilizing the rapid Turbo FISH hybridization protocol of Shaffer *et al* [16], the cells were fixed in pre-chilled methanol (-20°C) for 10 min, hybridized for 5 min, washed twice with 10% formamide in 2× SSC solution, and mounted for imaging.

### Fluorescence imaging and analysis

Cells were imaged with a Zeiss Axiovert 200M inverted, wide-field fluorescence microscope using a 100× oil immersion objective. Images were captured with a CoolSNAP HQ camera (Photometrics) using OPENLAB software (Perkin-Elmer). For each fluorescence channel, a 2-sec exposure was used to acquire 16–20 z-sections, 0.2 μm apart from each other. The z-stack images were then analyzed, utilizing a custom computer program written in MATLAB software (MathWorks) that identifies spot-like signals in each image, and determines their three-dimensional coordinates. This program, described in detail in Batish *et al*., 2012 [17], then identifies spots that have a counterpart within a 250-nm distance in the other channel. Spots meeting this criterion are classified as co-localized, representing fused transcripts. This computer program is freely available from our laboratory to all who would like to use it.

### Comparison of Fusion FISH imaging to real-time PCR

K562 cells were grown to 80% confluence. Some of the cells were fixed on coverslips and analyzed by our single-molecule RNA FISH method for the detection of fusion transcripts (“Fusion FISH”). The remaining cells were trypsinized and collected in 10 mL of 1× PBS. 100 μL of cells were mixed with 100 μL of trypan blue to count the number of cells, utilizing a hemocytometer. Total RNA was isolated from these cells utilizing TRIzol reagent (Invitrogen). The average number of fusion transcripts per cell isolated from these cells was then determined by quantitative reverse-transcriptase (qRT) real-time PCR. To prepare a standard curve for analysis of the PCR results, a plasmid containing the full-length BCR-ABL gene sequence under the control of a bacteriophage T7 promoter (pcDNA3, Addgene plasmid 27481) was linearized and used as a template for *in vitro* transcription by T7 RNA polymerase. The *in vitro*-transcribed RNA was then treated with deoxyribonuclease (New England Biolabs) and purified using a Zymo RNA purification kit (Zymo Research), and the concentration of the transcripts was determined with a NanoDrop spectrophotometer (Thermo Scientific). Purified RNA was serially diluted, and the resulting dilutions, along with three different dilutions of total cellular RNA from the K562 cells, were used as templates in a qRT- PCR reaction containing the following primers: 5′-GAAGTGTTTCAGAAGCTTCTCC-3′ and 5′-GTTTGGGCTTCACACCATTCC-3′. Amplification was carried out with a Qiagen one-step RT-PCR kit using SYBR Green as indicator.

## Results

### Design of Fusion FISH

**Figure 1:**
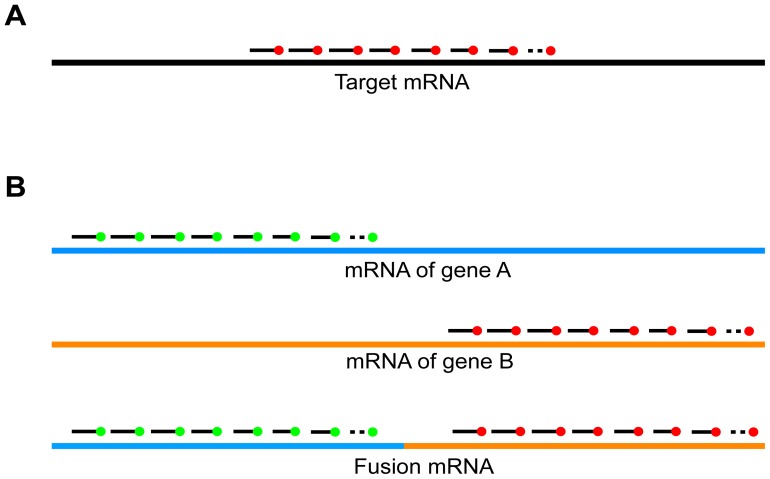
Principle of Fusion FISH imaging. (A) In single-molecule FISH, a set of approximately 50 oligonucleotides, each labeled with one fluorophore of the same color, bind to a segment of a target mRNA molecule, rendering it highly fluorescent. (B) Schematic representation of Fusion FISH for the detection of recombinant mRNAs transcribed from a fused gene created by a chromosomal translocation or rearrangement, in which two sets of Fusion FISH probes are utilized simultaneously, each set labeled with a differently colored fluorophore, and each set specific for a section of the fusion transcript that was encoded by one of the original partner genes. Fusion mRNAs are identified in microscope images of fixed cells by the co-localization of two differently colored spots arising from the binding of the two probe sets to the same target molecule.

In order to detect single molecules of fusion transcripts, we utilized single-molecule FISH (sm-FISH) probes, which were invented in our laboratory [15]. In this method, a set of about 50 singly labeled oligonucleotide probes, each about 20 nucleotides in length, bind to adjacent positions on a selected region of a target mRNA in fixed cells (Fig. 1a). The simultaneous binding of so many probes to each target mRNA molecule causes the target mRNA molecules to each appear as a diffraction-limited spot in a fluorescence microscope [15]. All molecules of target mRNA that are present in a cell can be located by acquiring 10 to 30 optical slices, each 0.2 μm thick, followed by image analysis of the resulting z-stack, using a custom image analysis computer program. These automated analyses provide detailed views of the behavior of individual cells [14], [15]. The single-molecule sensitivity of this method has previously been confirmed [15], [17], [18]. Most importantly, multiple mRNAs species can be imaged simultaneously, utilizing different probe sets, each labeled with a differently colored fluorophore; and computer algorithms can rapidly identify each mRNA species in a cell, determine the location of each target mRNA molecule within a cell, and count the number of molecules of each mRNA species that are present in the cell. Moreover, many cells can be analyzed simultaneously within the same tissue section [19].

To image gene fusion transcripts, we designed one set of probes that bind to the 5′ portion of the fusion mRNA, and a second set of probes (labeled with a differently colored fluorophore) that bind to the 3′ portion of the fusion mRNA (Fig. 1b). Cells are imaged with respect to each set of probes in the corresponding fluorescence channel. Fused transcripts give rise to spots that appear in both channels, whereas mRNAs produced by the normal alleles are visible in only one channel.

### Detection of BCR-ABL transcripts in chronic myeloid leukemia

**Figure 2:**
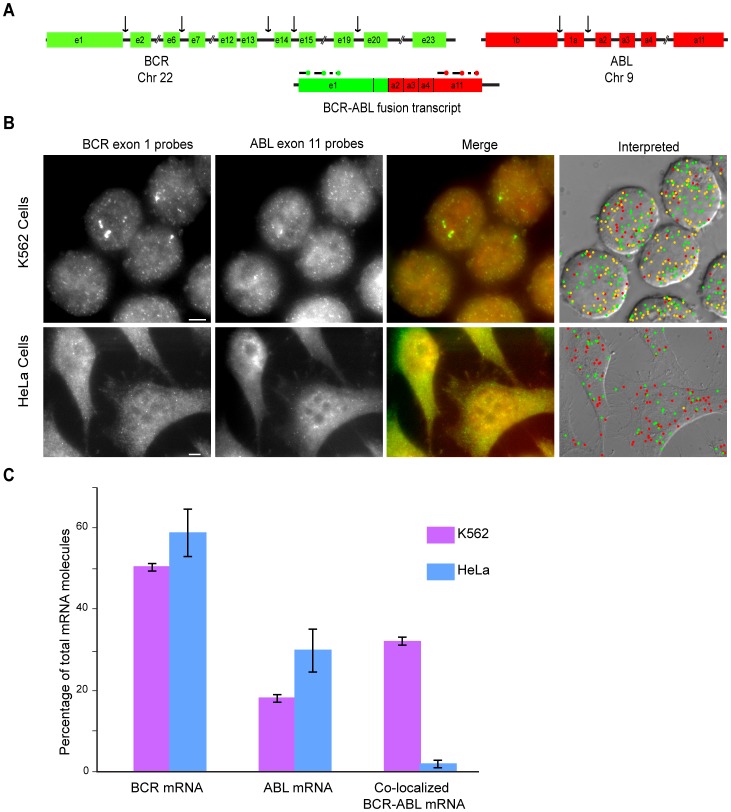
Detection of BCR-ABL fusion transcripts by Fusion FISH imaging. (A) Representation of the organization of the BCR and ABL genes, with arrows indicating reported break point sites (not drawn to scale). The resulting fusion transcripts always contain exon 1 of BCR and exon 11 of ABL. (B) Gray-scale fluorescence images (four left-hand panels) are merged images (z-stacks) obtained from each fluorescence color channel. Images arising from the BCR probe set (rendered in green), and images arising from the ABL probe set (rendered in red), were merged to obtain the images shown in the third set of panels, and the spots in those merged images were analyzed by computer alogrithm to identify spots arising from the BCR probe set alone, spots arising from the ABL probe set alone, and spots arising from the co-localization of both probe sets. The right-hand panels depict the identity of each algorithmically identified mRNA molecule laid over a differential interference contrast (DIC) image of the cells. Green circles identify BCR exon 1; red circles identify ABL exon 11; and yellow circles identify co-localized BCR exon 1 and ABL exon 11 (fusion transcripts). The scale bar is 5 μm long. (C) The spots identified in each Fusion FISH image were counted to obtain the percentage of each mRNA species that were present among all labeled transcripts in the image. The heights of the bars represent the average percentage obtained from at least 100 cells from each cell line, with the 95% confidence interval indicated by error bars.

In order to confirm that Fusion FISH can be used for the detection of gene fusion transcripts, we imaged the mRNA molecules transcribed from “Philadelphia chromosomes,” which contain the well-characterized gene fusion that occurs in chronic myeloid leukemia. Philadelphia chromosomes arise as a result of a translocation between the long arm of chromosome 9 and the long arm of chromosome 22, which is denoted t(9;22)(q34;q11). This chromosomal switch fuses part of the Breakpoint Cluster Region (BCR) gene of chromosome 22 with the Abelson (ABL) gene of chromosome 9, resulting in a fused gene that is transcribed, and that produces a functional protein [5]. Two different probe sets were designed; one set was labeled with tetramethylrhodamine (TMR), and was specific for exon 11 of ABL mRNA, which is located at the 3′ end of ABL mRNA; and the other probe set was labeled with Alexa Fluor 594, and was specific for exon 1 of BCR mRNA, which is located at the 5′ end of BCR mRNA (Fig. 2a). HeLa cells were used as representative “normal” cells, and K562 cells, which are immortalized CML cells, were used as representative cancer cells. Examination of the Fusion FISH images obtained from both cell types after hybridization with the two probe sets confirmed that distinct spots for ABL exon 11 and for BCR exon 1 could be seen in the respective fluorescence channels (Fig. 2b, left-hand panels). When we merged the images, we found many spots of BCR and ABL co-localized with each other in the K562 cells, while almost no co-localization of spots was seen in the HeLa cells, because the two genes are not fused (Fig. 2b, middle panels).

### Molecular co-localization analysis

Although co-localized spots in a merged image are visibly distinct from one target molecule to another, whether these spots arise from the same transcript molecule, or whether they arise from different transcript molecules, needs to be determined. Since the length of target mRNAs is much smaller than the diffraction limit, it was expected that diffraction-limited spots emanating from the same object in two different channels would overlap precisely. However, it turns out that spectral shifts, misalignments, and other experimental factors result in a finite separation between the two spots [17]. To obtain an empirical measure of these distances, we previously carried out a control experiment in which two halves of the same mRNA were imaged using two sets of differently colored sm-FISH probes, and we measured the distribution of distances of the centers of nearest neighbor pairs in three dimensions [17]. The distribution of these distances indicated that two spots can be located with a precision of about 110 nm (more refined methods of locating the centers of these spots improve the precision to about 30 nm [20]). The full span of the distribution provides an empirical limit (250 nm) below which two spots can be considered to be arising from the same molecule; and if the distance between the centers of two spots from different channels is more than 250 nm, the spots are classified as arising from distinct mRNA molecules.

Utilizing the 250 nm limit for co-localization, Fusion FISH images from K562 cells and from HeLa cells were compared. The results are shown in the right-hand panels of Fig. 2b, where green circles identify the locations of BCR target sequences, red circles identify the locations of ABL target sequences, and yellow circles identify the locations of co-localized BCR and ABL target sequences. In the images of the K562 cells, about 30% of the spots were co-localized, identifying the presence of BCR-ABL fusion transcripts, whereas almost no co-localized spots were seen in HeLa cells (Fig. 2c).

### Comparison of Fusion FISH imaging to real-time PCR

In order to establish the accuracy of the fusion transcript counts obtained by Fusion FISH imaging, we performed quantitative reverse-transcription PCR (qRT-PCR) on the total RNA isolated from K562 cells. Primers were selected from sequences on either side of the junction point of the BCR-ABL fusion. Fusion transcripts were obtained by transcription from a plasmid containing the fused BCR-ABL gene under the control of a T7 promoter. Serial dilutions of this mRNA were used to obtain a standard curve, which was used to determine the number of fusion transcripts in the total RNA (Fig. S1, left-hand panel). Dividing the number of fusion mRNA molecules determined by qRT-PCR by the number of cells from which the total RNA was obtained, we determined that the number of fusion transcripts per cell was 39±10. This measurement was very close to the 50±5 co-localized spots per cell obtained by Fusion FISH imaging (Fig. S1, right-hand panel), supporting the view that Fusion FISH imaging accurately identifies all of the fusion transcripts present in the cells that express them.

### Detection of EWSR1-FLI1 transcripts in Ewing's sarcoma

Although, the foregoing experiments demonstrate the efficacy of transcript detection by Fusion FISH, there exist effective PCR-based assays for detecting cells possessing the BCR-ABL fusion. A method for detecting fusion transcripts is more acutely needed for solid and soft-tissue tumors, where the distribution of cancerous cells among healthy cells could potentially be indicative of the stage and malignant potential of the tumor.

**Figure 3:**
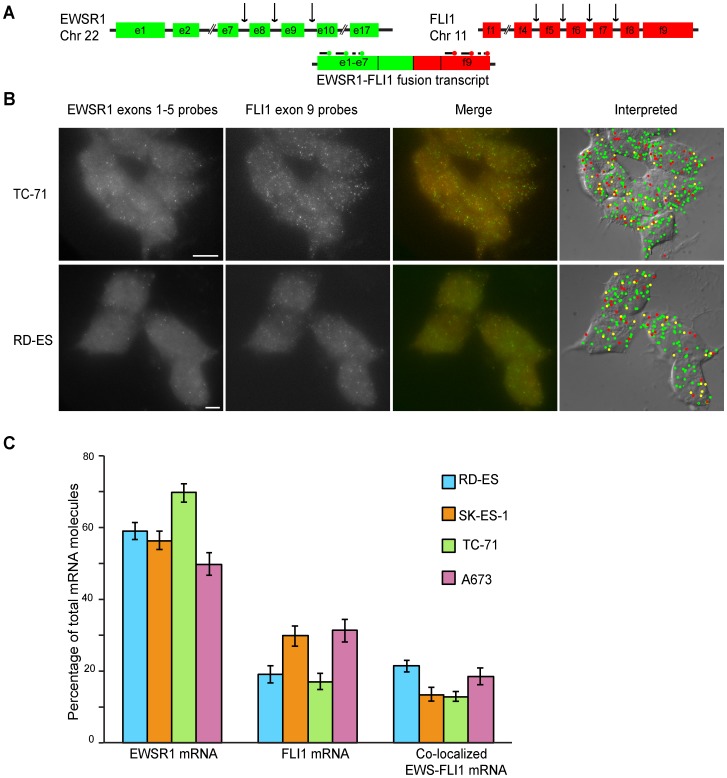
Detection of EWSR1-FLI1 fusion transcripts by Fusion FISH imaging. (A) Representation of the organization of the EWSR1 and FLI1 genes, with arrows indicating reported break point sites (not drawn to scale). The resulting fusion transcripts always contain exons 1 to 5 of EWSR1 and exons 8 to 9 of FLI1. (B) Representative images arising from Type 1 “7/6” (TC-71) and Type 2 “7/5” (RD-ES) fusions. Gray-scale fluorescence images in the four left-hand panels are merged images (z-stacks) obtained from each fluorescence color channel. Images arising from the EWSR1 probe set (rendered in green), and images arising from the FLI1 probe set (rendered in red), were merged to obtain the images shown in the third set of panels, and the spots in those merged images were analyzed by computer to identify spots arising from the EWSR1 probe set alone, spots arising from the FLI1 probe set alone, and spots arising from the co-localization of both probe sets. The right-hand panels depict the identity of each algorithmically identified mRNA molecule laid over a DIC image of the cells. Green circles identify EWSR1 exons 1 to 5; red circles identify FLI1 exon 9; and yellow circles identify co-localized EWSR1 exons 1 to 5 and FLI1 exon 9 (fusion transcripts). The scale bar is 5 μm long. (C) The spots identified in each Fusion FISH image were counted to obtain the percentage of each mRNA species that were present among all labeled transcripts in the image. The heights of the bars represent the average percentage obtained from at least 100 cells from each cell line, with the 95% confidence interval indicated by error bars.

Well-characterized examples of such tumors arising from fused genes include sarcomas, such as myxoid liposarcoma and Ewing's sarcoma [21]. Ewing's sarcoma is a malignant round-cell tumor affecting bones and soft tissue. About 90% of reported cases have a translocation between the EWSR1 gene of chromosome 22 and the FLI1 gene of chromosome 11, denoted as t(11;22)(q24;q12), which results in the synthesis of EWSR1-FLI1 chimeric mRNA [21], [22]. However, several different breakpoints in the participating genes are involved (Fig. 3a). The resulting fusions are categorized into different types, based on the number of exons of each fusion partner that are present in the chimeric mRNA [22]. It is suspected that different fusion types exert differential influence on disease progression and relapse [23].

To demonstrate the ability of Fusion FISH to detect fusion transcripts in Ewing's sarcomas, two differently colored probe sets were prepared, one set, labeled with Alexa Fluor 594, was specific for the 5′ region (exons 1 to 5) of EWSR1 mRNA, and the other set, labeled with TMR, was specific for the 3′ region (exon 9) of FLI1 mRNA. Four different cell lines were tested, each containing a common chromosomal translocation, to image the resulting EWSR1-FLI1 fusion mRNAs (Fig. 3b). Cell lines TC-71 and A673 harbor a Type1 “7/6” fusion, while cell lines RD-ES and SK-ES-1 harbor a Type 2 “7/5” fusion. The numbers in quotes correspond to the exons of EWSR1 and FLI1, respectively, that are present in the fused genes. The Fusion FISH probes that were used were designed to target regions of the fusion transcripts that are present in the majority of reported EWS-FLI1 Ewing's sarcoma fusion types [23]. We imaged each of the four cell lines. Representative images are presented for two of the cell lines in Fig. 3b. The color-coded merged images (right-hand panels) show that fused transcripts can be detected in both fusion types. A co-localization analysis was performed on images obtained from each of the four cell lines. The results indicate that 15-20% of the identified transcripts in these cell lines are fusion transcripts (Fig. 3c). However, there was no significant difference in the abundance of fusion transcripts among different cell types (which express distinct fusion mRNAs). This result is in agreement with previously published results which conclude that no significant difference is seen in the pathology of disease outcomes arising from these fusions [23]. It will be interesting to image cell lines harboring rare and more variant fusion transcript types, such as “7/8” and “10/4”, to see their effect on disease progression [22], [23].

Interestingly, in normal cells, there is no expression of FLI1 mRNA [24]. We tested our control cell line, HeLa, for the expression of EWSR1 mRNA and FLI1 mRNA, and we found that they express EWSR1 mRNA, but they do not express FLI1 mRNA; nor, as expected, did they express any fusion transcripts (Fig. S2).

## Discussion

The identification of chromosomal translocations that result in transcribed fused genes provides an attractive platform for developing cancer-specific assays, particularly in the case of solid tumors. Currently, most of the early screening for solid tumors is usually done by determining the level of surrogate biomarkers, for example, prostate specific antigen in the serum of prostate cancer patients. These biomarkers are tissue specific, but not necessarily cancer specific, and therefore often lead to over diagnosis. Accordingly, one-third of patients showing biomarker-based prostate cancer indications do not have cancer [25]. The discovery of gene fusion events that underlie solid tumors, including prostate cancer, has motivated the development of more sensitive and accurate molecular diagnostic assays [25], [26]. Using Fusion FISH imaging, it will become routine to identify tumor cells based on the number of fusion transcripts that are detected in each cell. This precise molecular marker-based identification of cancer cells will enable the use of “digital pathology slides.” In these digital representations of tissue sections, cancer cells identified by Fusion FISH will be displayed in a different color than normal cells. These digital views will greatly improve cancer diagnosis, and will help physicians to make rational decisions concerning the use of targeted therapies [27].

Traditional DNA FISH provides contextual information concerning the tumor environment. However, there are inherent disadvantages to imaging DNA fusions. First, there are only two loci to image in each cell, and these can be easily missed, leading to false negative results. Second, DNA is so compactly packed within the chromatin that there is a fair chance that DNA FISH probes will indicate co-localization just because of the chance proximity of the target regions. And finally, many gene fusion events do not lead to a functional fusion transcript. DNA FISH cannot provide information as to which chromosomal translocations actively produce fusion transcripts. Fusion FISH imaging of mRNAs addresses these limitations, since mRNAs are more ubiquitous, and only fusion transcripts give rise to unique identifiable signals.

The results of the experiments presented in this paper demonstrate the ability of Fusion FISH to identify fusion transcripts in cultured cells. Clinical samples, on the other hand, especially those from solid tumors, are likely be formalin-fixed and paraffin-embedded (FFPE) or frozen tissue sections. Several groups have successfully used sm-FISH probes to view mRNAs in clinical samples [19], [28], [29]. We anticipate that we will be able to use Fusion FISH probes for clinical samples without significant problems. An interesting recent report by Tsugita *et al*. has shown that Ewing's sarcoma cells secrete EWSR1-FLI1 mRNAs in microvesicles released into blood [30]. We could employ Fusion FISH imaging to detect fusion transcripts in the serum of Ewing's sarcoma patients, potentially enabling sensitive diagnosis without the need for invasive procedures.

There are certain genes that are involved in several different cancers by virtue of having multiple fusion partners. One such example is the gene for anaplastic lymphoma kinase (ALK). The ALK gene is present on chromosome 2, encodes a tyrosine kinase, and is responsible for cell growth. The 3′ portion of the ALK gene is sometimes fused with the 5′ segment of the echinoderm microtubule-associated protein-like 4 (EML4) gene, which is associated with approximately 7% of non-small-cell lung cancer (NSCLC) cases [31], or it can associate with the 5′ region of the nucleophosmin (NPM) gene, found in approximately 60% of anaplastic large-cell lymphoma (ALCL) cases [32]. An independent study has recently shown the use of smFISH in detecting gene fusions underlying NSCLC [33]. Fusion FISH imaging can also be used in a unique manner, by utilizing two differently colored probe sets, one set specific for the 5′ end of a target mRNA, and the other set specific for the 3′ end of the same mRNA. For example, just probing the ALK mRNA alone, it will be possible to identify whether the ALK gene is intact and able to synthesize a complete mRNA (in which case the spots from both probe sets will be similar in number and will all co-localize), or whether the ALK gene has undergone rearrangement (in which case, the differently colored spots will not co-localize). This can also be used as a tool to identify new gene rearrangements based on sequencing data.

A desirable feature of any diagnostic assay is that it should achieve results in the shortest possible time. Conventional sm-FISH requires an overnight incubation with probes. However, the entire process can be accomplished in less than 30 minutes by utilizing an alternative approach for fixation and hybridization called “Turbo FISH” [15]. Recently, we tested Fusion FISH probes under Turbo FISH conditions, and we obtained similar results. Therefore, the use of Fusion FISH probes in combination with Turbo FISH should enable accurate, single-molecule imaging, and subsequent diagnosis, in less time than it takes to perform one-step PCR assays.

An inherent limitation of Fusion FISH is that it requires a long stretch of mRNA to serve as a target sequence (600–800 nucleotides) for each set of probes. In certain gene fusions, for example TMRPSS2-ERG in prostate cancer [26], only one exon of an mRNA juxtaposes with a segment of the partner mRNA, in which case it will not be possible to identify fusion transcripts using conventional Fusion FISH imaging. In such cases, we could image the short RNA segment utilizing branched DNA probes that, instead of being labeled with one fluorophore, are labeled with a larger number of fluorophores, thereby enabling the imaging of single fusion mRNA molecules, even if one target segment is quite short [34]. Another alternative is to use a recent approach called FISH STICs, wherein a single unlabeled probe targeting 50 nt target region is hybridized to the target. This probe is designed to have a repeat sequence at the 3′ end to which a set of three secondary unlabled probes bind in secondary hybridization. Each secondary probe provides a binding site for 15 dye-labeled probes in a tertiary hybridization, hence giving a signal equivalent to 45 linear probes [35]. This approach can be used in combination with Fusion FISH to enable imaging of shorter fragments of RNAs and might also enable one to distinguish between different types of fusion transcripts originating due to different breakpoints in the same gene.

In summary, Fusion FISH imaging of individual mRNA molecules should provide a powerful new tool for the diagnosis, management, and exploration of cancers that occur via gene fusions.

## Supporting Information

Figure S1 **Comparison of Fusion FISH with qRT-PCR.** (Left-hand panel) Standard curve obtained using serial dilutions of full-length *in vitro*-transcribed BCR-ABL mRNA molecules as templates for qRT-PCR. The result obtained by using total RNA isolated from K562 cells is represented by a green dot. The equation of the fitted line was used to calculate mRNA molecules per cell. (Right-hand panel) Comparison of fusion mRNA molecules per cell determined by Fusion FISH imaging to mRNA copy number per cell determined by real-time PCR. Error bars represent 95% confidence intervals.
(TIF)

Figure S2 **Demonstration of the specificity of Fusion FISH probes.** HeLa cells, which are known to not express FLI mRNA, were imaged using EWSR1 exon 1 to 5 probes labeled in one color and FLI1 exon 9 probes labeled in a different color. No spots were seen in the color used to label FLI1 probes, implying that no fusion transcripts were synthesized in HeLa cells, and indicating that the Fusion FISH imaging is highly specific for its target sequence. The top panels were obtained by merging three-dimensional images (z-stacks). In the image on the lower right, algorithmically identified molecules are laid over a DIC image of the cells. Green circles identify EWSR1 exons 1 to 5; No red circles (identifying FLI1 exon 9); and no yellow circles (identifying fusion transcripts) are present. The scale bar is 5 μm long.
(TIF)

Table S1 **The sequences of Fusion FISH probes used in the study.**
(XLS)
