# Supplementary figures and images for: Fusion FISH Imaging: Single-Molecule Detection of Gene Fusion Transcripts In Situ
Source: PLoS One. 2014 Mar 27;9(3):e93488. doi: 10.1371/journal.pone.0093488 (PMC3968151; doi:10.1371/journal.pone.0093488)

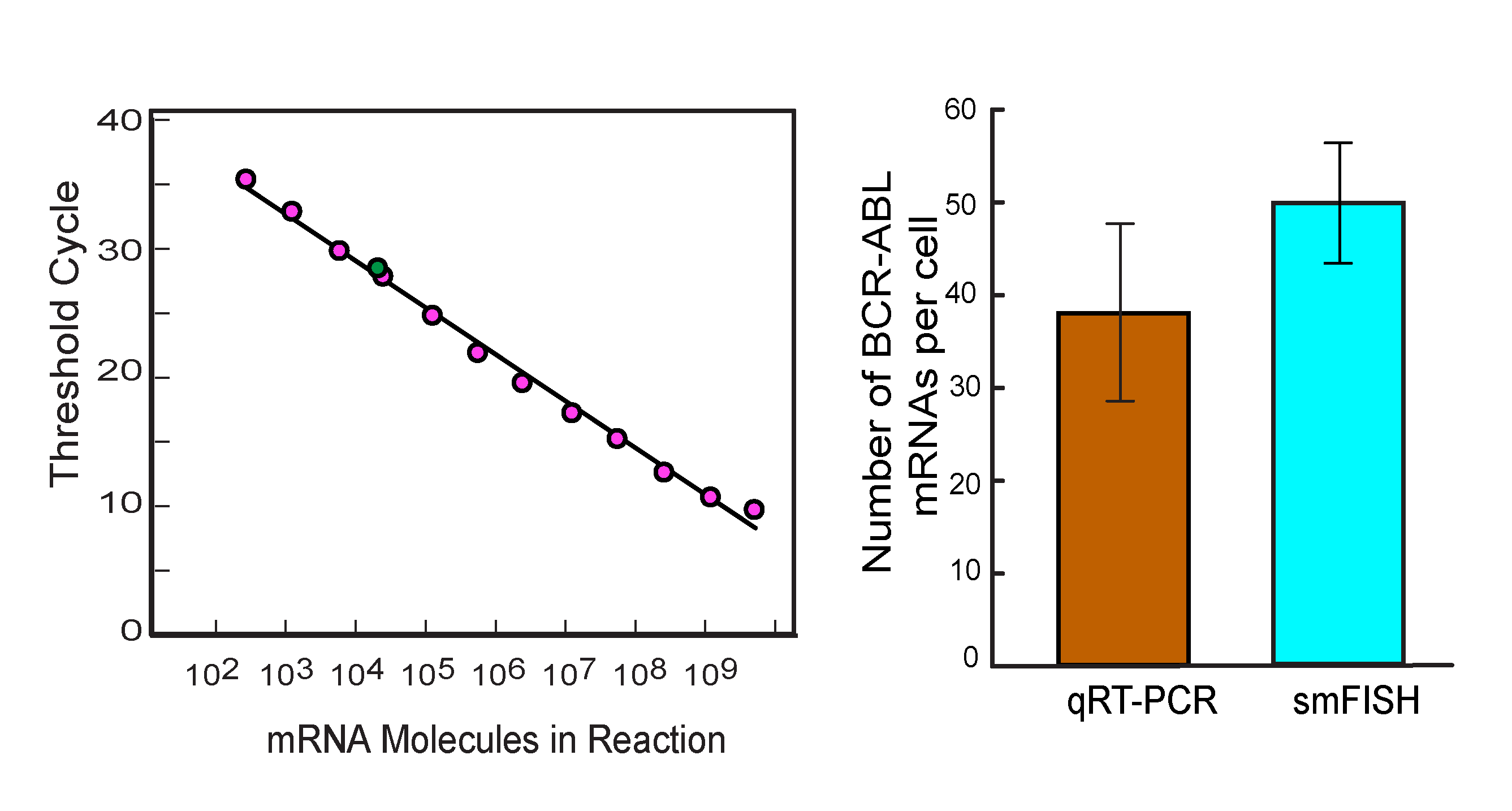

Supplement: Figure S1 — Comparison of Fusion FISH with qRT-PCR. (Left-hand panel) Standard curve obtained using serial dilutions of full-length in vitro-transcribed BCR-ABL mRNA molecules as templates for qRT-PCR. The result obtained by using total RNA isolated from K562 cells is represented by a green dot. The equation of the fitted line was used to calculate mRNA molecules per cell. (Right-hand panel) Comparison of fusion mRNA molecules per cell determined by Fusion FISH imaging to mRNA copy number per cell determined by real-time PCR. Error bars represent 95% confidence intervals. (TIF) [file pone.0093488.s001.tif]

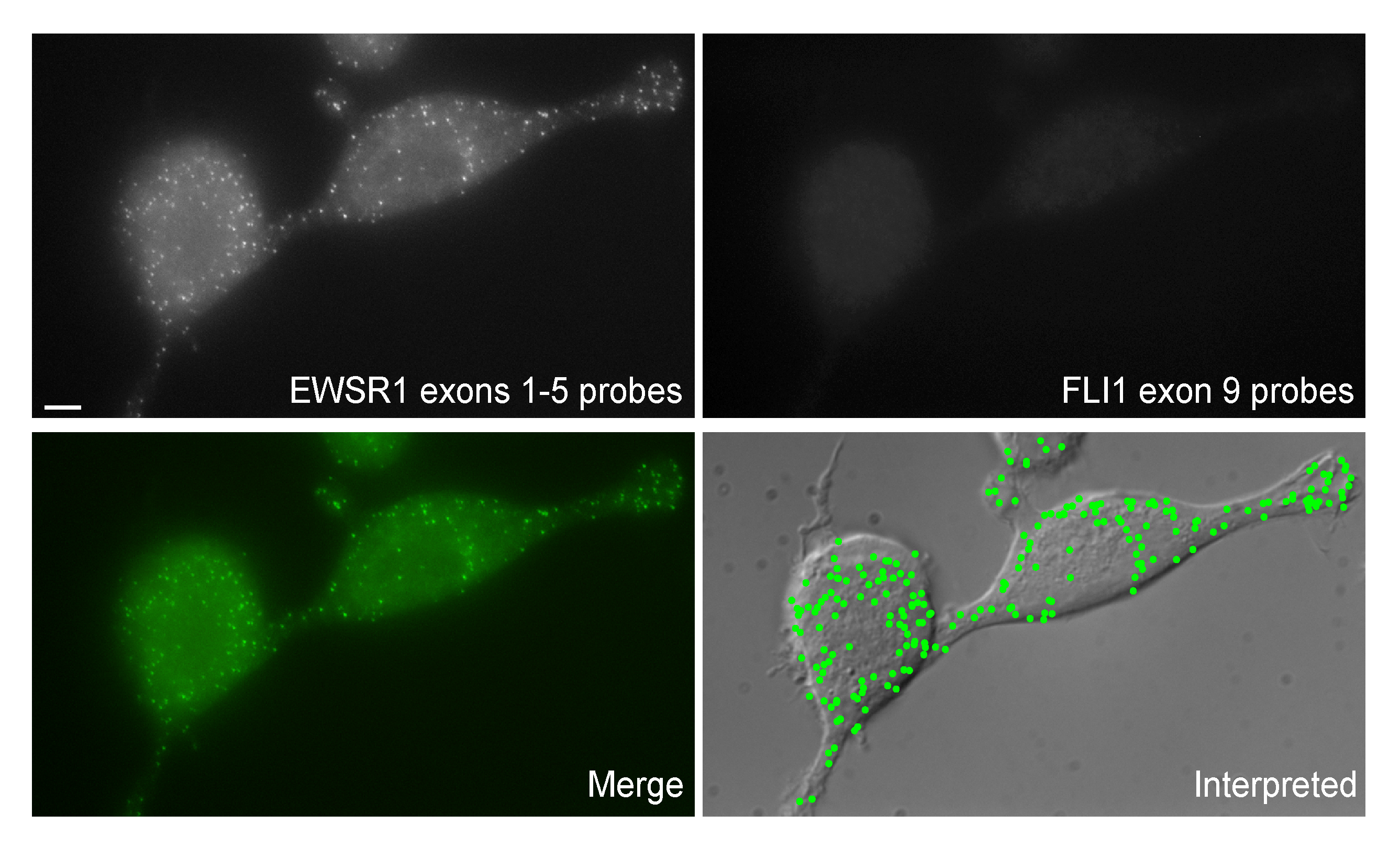

Supplement: Figure S2 — Demonstration of the specificity of Fusion FISH probes. HeLa cells, which are known to not express FLI mRNA, were imaged using EWSR1 exon 1 to 5 probes labeled in one color and FLI1 exon 9 probes labeled in a different color. No spots were seen in the color used to label FLI1 probes, implying that no fusion transcripts were synthesized in HeLa cells, and indicating that the Fusion FISH imaging is highly specific for its target sequence. The top panels were obtained by merging three-dimensional images (z-stacks). In the image on the lower right, algorithmically identified molecules are laid over a DIC image of the cells. Green circles identify EWSR1 exons 1 to 5; No red circles (identifying FLI1 exon 9); and no yellow circles (identifying fusion transcripts) are present. The scale bar is 5 μm long. (TIF) [file pone.0093488.s002.tif]
